# Deep immune profiling of COVID-19 patients reveals distinct immunotypes with therapeutic implications

**DOI:** 10.1126/science.abc8511

**Published:** 2020-07-15

**Authors:** Divij Mathew, Josephine R. Giles, Amy E. Baxter, Derek A. Oldridge, Allison R. Greenplate, Jennifer E. Wu, Cécile Alanio, Leticia Kuri-Cervantes, M. Betina Pampena, Kurt D’Andrea, Sasikanth Manne, Zeyu Chen, Yinghui Jane Huang, John P. Reilly, Ariel R. Weisman, Caroline A. G. Ittner, Oliva Kuthuru, Jeanette Dougherty, Kito Nzingha, Nicholas Han, Justin Kim, Ajinkya Pattekar, Eileen C. Goodwin, Elizabeth M. Anderson, Madison E. Weirick, Sigrid Gouma, Claudia P. Arevalo, Marcus J. Bolton, Fang Chen, Simon F. Lacey, Holly Ramage, Sara Cherry, Scott E. Hensley, Sokratis A. Apostolidis, Alexander C. Huang, Laura A. Vella, The UPenn COVID Processing Unit, Michael R. Betts, Nuala J. Meyer, E. John Wherry

**Affiliations:** 1 Institute for Immunology, University of Pennsylvania Perelman School of Medicine, Philadelphia, PA, USA.; 2 Department of Systems Pharmacology and Translational Therapeutics, University of Pennsylvania Perelman School of Medicine, Philadelphia, PA, USA.; 3 Parker Institute for Cancer Immunotherapy, University of Pennsylvania Perelman School of Medicine, Philadelphia, PA, USA.; 4 Department of Pathology and Laboratory Medicine, University of Pennsylvania Perelman School of Medicine, Philadelphia, PA, USA.; 5 Department of Microbiology, University of Pennsylvania Perelman School of Medicine, Philadelphia, PA, USA.; 6 Division of Translational Medicine and Human Genetics, University of Pennsylvania Perelman School of Medicine, Philadelphia, PA, USA.; 7 Division of Pulmonary, Allergy and Critical Care Medicine, Center for Translational Lung Biology, Lung Biology Institute, Department of Medicine, University of Pennsylvania Perelman School of Medicine, Philadelphia, PA, USA.; 8 Division of Gastroenterology, Department of Medicine, University of Pennsylvania Perelman School of Medicine, Philadelphia, PA, USA.; 9 Center for Cellular Immunotherapies, University of Pennsylvania Perelman School of Medicine, Philadelphia, PA, USA.; 10 Department of Microbiology, Thomas Jefferson University, Philadelphia, PA, USA.; 11 Division of Rheumatology, Department of Medicine, University of Pennsylvania Perelman School of Medicine, Philadelphia, PA, USA.; 12 Division of Hematology and Oncology, Department of Medicine, University of Pennsylvania Perelman School of Medicine, Philadelphia, PA, USA.; 13 Division of Infectious Disease, Department of Pediatrics, Children’s Hospital of Philadelphia, Philadelphia, PA, USA.; 14 Division of Pulmonary and Critical Care Medicine, Department of Medicine, University of Pennsylvania Perelman School of Medicine, Philadelphia, PA, USA.

## Abstract

Coronavirus disease 2019 (COVID-19) is currently a global pandemic, but human immune responses to the virus remain poorly understood. We used high-dimensional cytometry to analyze 125 COVID-19 patients and compare them with recovered and healthy individuals. Integrated analysis of ~200 immune and ~50 clinical features revealed activation of T cell and B cell subsets in a proportion of patients. A subgroup of patients had T cell activation characteristic of acute viral infection and plasmablast responses reaching >30% of circulating B cells. However, another subgroup had lymphocyte activation comparable with that in uninfected individuals. Stable versus dynamic immunological signatures were identified and linked to trajectories of disease severity change. Our analyses identified three immunotypes associated with poor clinical trajectories versus improving health. These immunotypes may have implications for the design of therapeutics and vaccines for COVID-19.

## Immune profiling of COVID-19 patients

Coronavirus disease 2019 (COVID-19) has affected millions of people globally, yet how the human immune system responds to and influences COVID-19 severity remains unclear. Mathew *et al.* present a comprehensive atlas of immune modulation associated with COVID-19. They performed high-dimensional flow cytometry of hospitalized COVID-19 patients and found three prominent and distinct immunotypes that are related to disease severity and clinical parameters. Arunachalam *et al.* report a systems biology approach to assess the immune system of COVID-19 patients with mild-to-severe disease. These studies provide a compendium of immune cell information and roadmaps for potential therapeutic interventions.

*Science*, this issue p. eabc8511, p. 1210

## Abstract (structured)

**INTRODUCTION:**

Many patients with coronavirus disease 2019 (COVID-19), caused by severe acute respiratory syndrome coronavirus 2 (SARS-CoV-2) infection, present with severe respiratory disease requiring hospitalization and mechanical ventilation. Although most patients recover, disease is complex and case fatality can be as high as 10%. How human immune responses control or exacerbate COVID-19 is currently poorly understood, and defining the nature of immune responses during acute COVID-19 could help identify therapeutics and effective vaccines.

**RATIONALE:**

Immune dysregulation during SARS-CoV-2 infection has been implicated in pathogenesis, but currently available data remain limited. We used high-dimensional cytometry to analyze COVID-19 patients and compare them with recovered and healthy individuals and performed integrated analysis of ~200 immune features. These data were combined with ~50 clinical features to understand how the immunology of SARS-CoV-2 infection may be related to clinical patterns, disease severity, and progression.

**RESULTS:**

Analysis of 125 hospitalized COVID-19 patients revealed that although CD4 and CD8 T cells were activated in some patients, T cell responses were limited in others. In many patients, CD4 and CD8 T cell proliferation (measured by KI67 increase) and activation (detected by CD38 and HLA-DR coexpression) were consistent with antiviral responses observed in other infections. Plasmablast (PB) responses were present in many patients, reaching >30% of total B cells, and most patients made SARS-CoV-2–specific antibodies. However, ~20% of patients had little T cell activation or PB response compared with controls. In some patients, responses declined over time, resembling typical kinetics of antiviral responses; in others, however, robust T cell and PB responses remained stable or increased over time. These temporal patterns were associated with specific clinical features. With an unbiased uniform manifold approximation and projection (UMAP) approach, we distilled ~200 immune parameters into two major immune response components and a third pattern lacking robust adaptive immune responses, thus revealing immunotypes of COVID-19: (i) Immunotype 1 was associated with disease severity and showed robust activated CD4 T cells, a paucity of circulating follicular helper cells, activated CD8 “EMRAs,” hyperactivated or exhausted CD8 T cells, and PBs. (ii) Immunotype 2 was characterized by less CD4 T cell activation, Tbet^+^ effector CD4 and CD8 T cells, and proliferating memory B cells and was not associated with disease severity. (iii) Immunotype 3, which negatively correlated with disease severity and lacked obvious activated T and B cell responses, was also identified. Mortality occurred for patients with all three immunotypes, illustrating a complex relationship between immune response and COVID-19.

**CONCLUSION:**

Three immunotypes revealing different patterns of lymphocyte responses were identified in hospitalized COVID-19 patients. These three major patterns may each represent a different suboptimal response associated with hospitalization and disease. Our findings may have implications for treatments focused on activating versus inhibiting the immune response.

## Teaser

Immune responses of COVID-19 patients are cataloged and compared with those of healthy individuals.

## Graphical abstract

**Figure:**
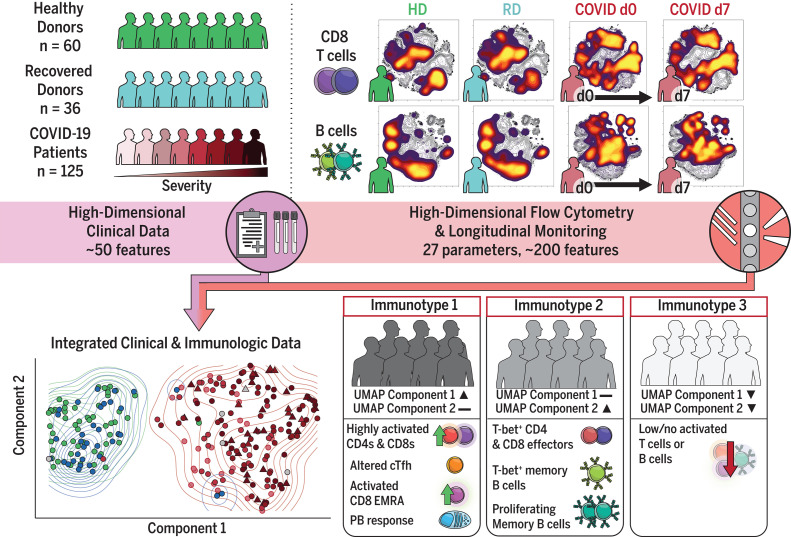
High-dimensional immune response analysis of COVID-19 patients identifies three immunotypes. Peripheral blood mononuclear cell immune profiling and clinical data were collected from 60 healthy donors (HDs), 36 recovered donors (RDs), and 125 hospitalized COVID-19 patients. High-dimensional flow cytometry and longitudinal analysis highlighted stability and fluctuations in the response. UMAP visualization distilled ~200 immune features into two dimensions and identified three immunotypes associated with clinical outcomes. cTfh, circulating T follicular helper cells; EMRA, a subset of effector memory T cells reexpressing CD45RA; d0, day 0.

The coronavirus disease 2019 (COVID-19) pandemic has, to date, caused >23 million infections resulting in more than 800,000 deaths. After infection with severe acute respiratory syndrome coronavirus 2 (SARS-CoV-2), COVID-19 patients can experience mild or even asymptomatic disease or can present with severe disease requiring hospitalization and mechanical ventilation. The case fatality rate can be as high as ~10% (*1*). Some severe COVID-19 patients display acute respiratory distress syndrome (ARDS), which reflects severe respiratory damage. In acute respiratory viral infections, pathology can be mediated by the virus directly, by an overaggressive immune response, or both (*2*–*4*). However, in severe COVID-19, the characteristics and role of the immune response, as well as how these responses relate to clinical disease features, remain poorly understood.

SARS-CoV-2 antigen-specific T cells have been identified in the central memory (CM), effector memory (EM), and CD45RA^+^ effector memory (EMRA) compartments (*5*), but the characteristics of these cells and their role in infection or pathogenesis remain unclear. Recovered individuals more often have evidence of virus-specific CD4 T cell responses than virus-specific CD8 T cell responses, though preexisting CD4 T cell responses to other coronaviruses also are found in a subset of people in the absence of SARS-CoV-2 exposure (*6*). Inflammatory responses—such as increases in interleukin-6 (IL-6)–producing or granulocyte-macrophage colony-stimulating factor (GM-CSF)–producing CD4 T cells in the blood (*7*) or decreases in immunoregulatory subsets such as regulatory T cells (T_reg_) or γδ T cells (*8*–*11*)—have been reported. T cell exhaustion (*12*, *13*) and increased inhibitory receptor expression on peripheral T cells have also been reported (*7*, *14*), though these inhibitory receptors are also increased after T cell activation (*15*). Although there is evidence of T cell activation in COVID-19 patients (*16*), some studies have found decreases in polyfunctionality (*12*, *17*) or cytotoxicity (*12*), but these changes have not been observed in other studies (*13*). How this activation should be viewed in the context of COVID-19 lymphopenia (*18*–*20*) remains unclear.

Most patients seroconvert within 7 to 14 days of infection, and increased plasmablasts (PBs) have been reported (*16*, *21*–*23*). However, the role of humoral responses in the pathogenesis of COVID-19 is still unclear. Whereas immunoglobulin G (IgG) levels reportedly drop slightly ~8 weeks after symptom onset (*24*, *25*), recovered patients maintain high spike protein–specific IgG titers (*6*, *26*). IgA levels also can remain high and may correlate with disease severity (*25*, *27*). Furthermore, neutralizing antibodies can control SARS-CoV-2 infection in vitro and in vivo (*4*, *28*, *29*). Indeed, convalescent plasma that contains neutralizing antibodies can improve clinical symptoms (*30*). However, not all patients that recover from COVID-19 have detectable neutralizing antibodies (*6*, *26*), which suggests a complex relationship between humoral and cellular response in COVID-19 pathogenesis.

Taken together, this previous work provokes questions about the potential diversity of immune responses to SARS-CoV-2 and the relationship of this diversity to clinical disease. However, many studies describe small cohorts or even single patients, thus limiting a comprehensive investigation of this diversity. The relationship of different immune response features to clinical parameters, as well as the changes in immune responses and clinical disease over time, remains poorly understood. Because potential therapeutics for COVID-19 patients include approaches to inhibit, activate, or otherwise modulate immune function, it is essential to define the immune response characteristics related to disease features in well-defined patient cohorts.

## Acute SARS-CoV-2 infection in humans results in broad changes in circulating immune cell populations

**Fig. 1:**
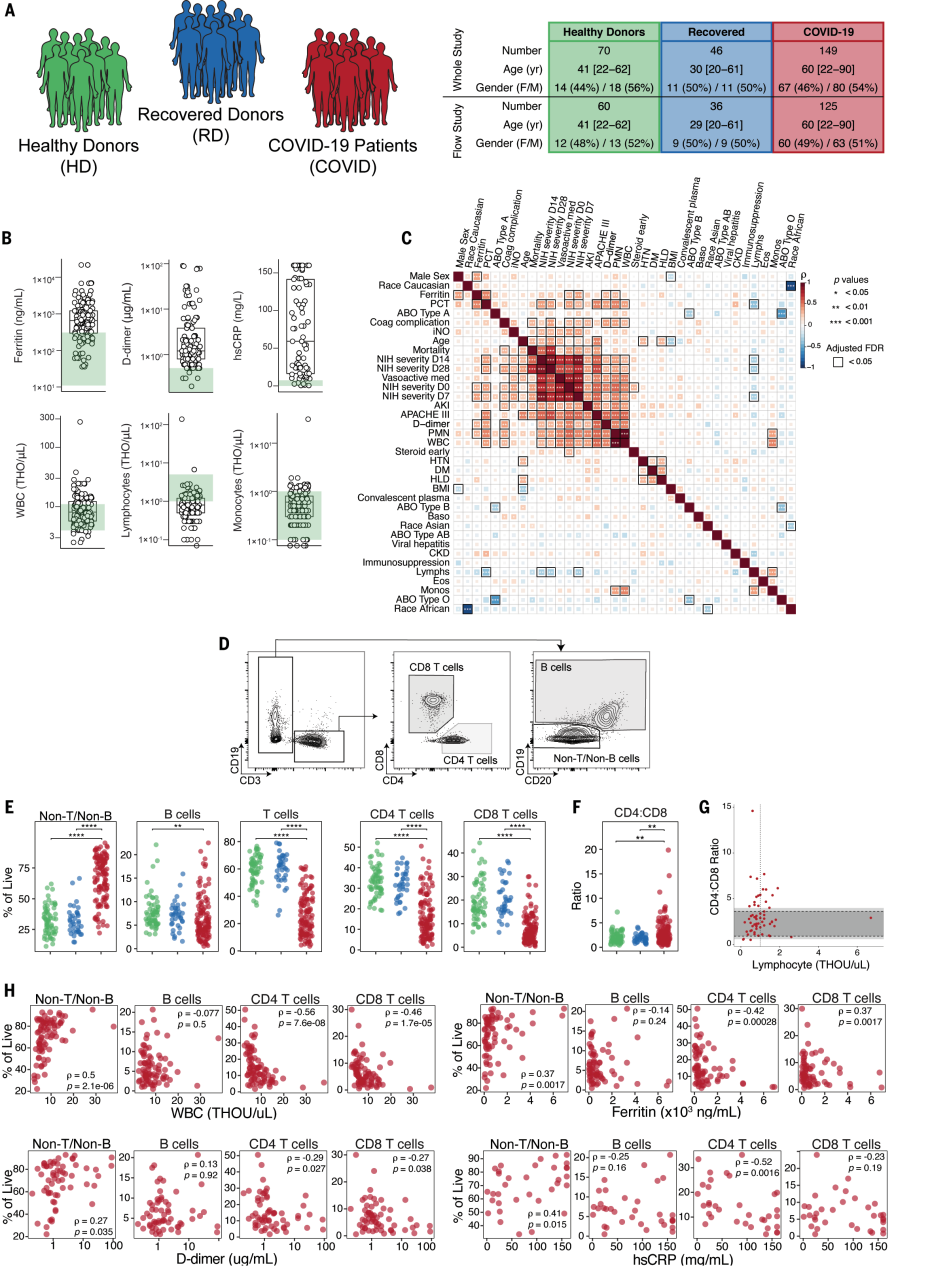
Clinical characterization of patient cohorts, inflammatory markers, and quantification of major immune subsets. (**A**) Overview of patient cohorts in our study, including HDs, RDs, and COVID-19 patients. (**B**) Quantification of key clinical parameters in COVID-19 patients. Each dot represents a COVID-19 patient; HD ranges are indicated in green. THO, ×1000. (**C**) Spearman correlation and hierarchical clustering of indicated features for COVID-19 patients. (**D**) Representative flow cytometry plots and (**E**) frequencies of major immune subsets. (**F**) Ratio of CD4 to CD8 T cells. (**G**) Spearman correlation of CD4:CD8 ratio and clinical lymphocyte count per patient. Dark and light gray shaded regions represent the clinical normal range and normal range based on study HDs, respectively. The vertical dashed line indicates the clinical threshold for lymphopenia. (**H**) Spearman correlations of indicated subsets with various clinical features. (E and F) Each dot represents an individual HDs (green), RDs (blue), or COVID-19 patient (red). Significance was determined by unpaired Wilcoxon test with Benjamini-Hochberg (BH) correction: *P < 0.05, **P < 0.01, ***P < 0.001, and ****P < 0.0001.

We conducted an observational study of hospitalized patients with COVID-19 at the University of Pennsylvania (UPenn IRB 808542) that included 149 adults with confirmed SARS-CoV-2 infection (i.e., COVID-19 patients) (Fig. 1A). Blood was collected at enrollment (typically ~24 to 72 hours after admission). Additional samples were obtained from patients who remained hospitalized on day 7 (D7). Blood was also collected from nonhospitalized patients who had recovered from documented SARS-CoV-2 infection [recovered donors (RDs); *n* = 46], as well as from healthy donors (HDs; *n* = 70) (UPenn IRB 834263) (Fig. 1A). Clinical metadata are available from the COVID-19 patients over the course of disease (table S1). Flow cytometry data from peripheral blood mononuclear cells (PBMCs), as well as clinical metadata, were collected from a subset of patients and donors: COVID-19 patients (*n* = 125), RDs (*n* = 36), and HDs (*n* = 60) (Fig. 1A and tables S2 to S4).

COVID-19 patients had a median age of 60 and were significantly older than HDs and RDs (median ages of 41 and 29, respectively), though the age distributions for all three cohorts overlapped (Fig. 1A and fig. S1A). For COVID-19 patients, median body mass index was 29 (range: 16 to 78), and 68% of these patients were African American (table S2). Comorbidities in COVID-19 patients were dominated by cardiovascular risk factors (83% of the cohort). Nearly 20% of patients suffered from chronic kidney disease, and 18% had a previous thromboembolic event. A subset of patients (18%) were immunosuppressed, and 7 and 6% of patients were known to have a diagnosis of cancer or a preexisting pulmonary condition, respectively. Forty-five percent of the patients were treated with hydroxychloroquine (HCQ), 31% with steroids, and 29% with remdesivir. Eighteen individuals died during their hospital stay or within 30 days of admission. The majority of the patients were symptomatic at diagnosis and were enrolled ~9 days after initiation of symptoms. Approximately 30% of patients required mechanical ventilation at presentation, with additional extracorporeal membrane oxygenation in four cases.

As has been reported for other COVID-19 patients (*31*), this COVID-19 cohort presented with a clinical inflammatory syndrome. C-reactive protein (CRP) was elevated in more than 90% of individuals and lactate dehydrogenase and D-dimer were increased in the majority, whereas ferritin was above normal in ~75% of COVID-19 patients (Fig. 1B and fig. S1B). Similarly, troponin and NT-proBNP were increased in some patients (fig. S1B). IL-6 levels, measured in a subset of patients, were normal in 5 patients, moderately elevated in 5 patients (6 to 20 pg/ml), and high in 31 patients (21 to 738 pg/ml) (fig. S1B). Although white blood cell (WBC) counts were mostly normal, individual leukocyte populations were altered in COVID-19 patients (Fig. 1B). A subset of patients had high polymorphonuclear leukocyte (PMN) counts (fig. S1B), as described previously (*8*, *32*) and in a companion study (*33*). Furthermore, approximately half of the COVID-19 patients were clinically lymphopenic (absolute lymphocyte count <1000/μl; Fig. 1B). By contrast, monocyte, eosinophil, and basophil counts were mostly normal (Fig. 1B and fig. S1B).

To examine potential associations between these clinical features, we performed correlation analysis (Fig. 1C and fig. S1C). This analysis revealed correlations between different COVID-19 severity metrics, as well as clinical features or interventions associated with more-severe disease (e.g., D-dimer, vasoactive medication) (Fig. 1C and fig. S1C). WBCs and PMNs also correlated with metrics of disease severity (e.g., APACHE III) as well as with IL-6 levels (Fig. 1C and fig. S1C). Other relationships were also apparent, including correlations between age or mortality and metrics of disease severity and many other correlations between clinical measures of disease, inflammation, and comorbidities (Fig. 1C and fig. S1C). Thus, COVID-19 patients presented with varied preexisting comorbidities, complex clinical phenotypes, evidence of inflammation in many patients, and clinically altered leukocyte counts.

To begin to investigate immune responses to acute SARS-CoV-2 infection, we compared PBMCs of COVID-19 patients, RDs, and HDs by using high-dimensional flow cytometry. We first focused on the major lymphocyte populations. B cell and CD3 T cell frequencies were decreased in COVID-19 patients compared with HDs or RDs, reflecting clinical lymphopenia, whereas the relative frequency of non-B and non-T cells was correspondingly elevated (Fig. 1, D and E). Although a numerical expansion of a non-B, non-T cell type is possible, loss of lymphocytes likely results in an increase in the relative frequency of this population. This non-B, non-T cell population is also probed in more detail in the companion study (*33*). Examining only CD3 T cells revealed preferential loss of CD8 T cells compared with CD4 T cells (Fig. 1, F and G, and fig. S1D); this pattern was reflected in absolute numbers estimated from the clinical data, where both CD4 and CD8 T cell counts in COVID-19 patients were lower than the clinical reference range, though the effect was more prominent for CD8 T cells (49 of 61 individuals with below-normal levels) than for CD4 T cells (38 of 61 individuals with below-normal levels) (fig. S1E). These findings are consistent with previous reports of lymphopenia during COVID-19 (*17*–*20*) but highlight a preferential impact on CD8 T cells.

We next asked whether the changes in these lymphocyte populations were related to clinical metrics (Fig. 1H). Lower WBC counts were associated preferentially with lower frequencies of CD4 and CD8 T cells and increased non-T, non-B cells, but not with B cells (Fig. 1H). These lower T cell counts were associated with clinical markers of inflammation, including ferritin, D-dimer, and high-sensitivity CRP (hsCRP) (Fig. 1H), whereas altered B cell frequencies were not. Thus, hospitalized COVID-19 patients present with a complex constellation of clinical features that may be associated with altered lymphocyte populations.

## SARS-CoV-2 infection is associated with CD8 T cell activation in a subset of patients

**Fig. 2:**
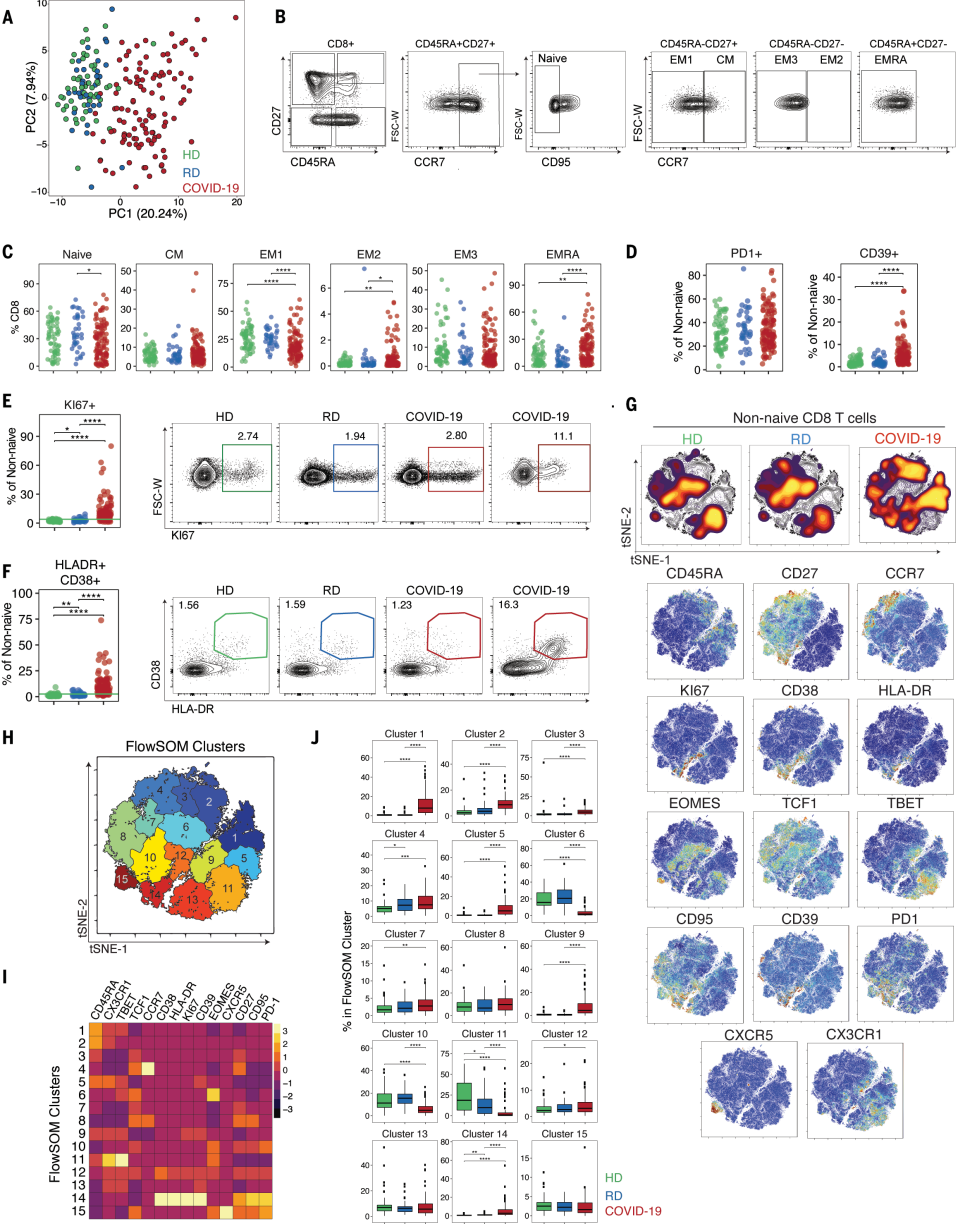
CD8 T cell subset skewing and activation patterns in COVID-19 patients and potential links to T cell–driven cytokines. (**A**) PCA of aggregated flow cytometry data. (**B**) Representative flow cytometry plots of the gating strategy for CD8 T cell subsets. (**C**) Frequencies of CD8 T cell subsets as indicated. (**D**) Frequencies of PD-1^+^ and CD39^+^ cells. Frequencies of (**E**) KI67^+^ and (**F**) HLA-DR^+^CD38^+^ cells and representative flow cytometry plots. The green line in the left panels denotes the upper decile of HDs. (**G**) (Top) Global viSNE projection of non-naïve CD8 T cells for all participants pooled, with non-naïve CD8 T cells from HDs, RDs, and COVID-19 patients concatenated and overlaid. (Bottom) viSNE projections of expression of the indicated proteins. (**H**) viSNE projection of non-naïve CD8 T cell clusters identified by FlowSOM clustering. (**I**) Mean fluorescence intensity (MFI) as indicated (column-scaled *z*-scores). (**J**) Percentage of non-naïve CD8 T cells from each cohort in each FlowSOM cluster. Boxes represent interquartile ranges (IQRs). (C, D, E, F, and J) Each dot represents an individual HDs (green), RDs (blue), or COVID-19 patient (red). Significance was determined by unpaired Wilcoxon test with BH correction: *P < 0.05, **P < 0.01, ***P < 0.001, and ****P < 0.0001.

We next applied high-dimensional flow cytometric analysis to further investigate lymphocyte activation and differentiation during COVID-19. We first used principal components analysis (PCA) to examine the general distribution of immune profiles from COVID-19 patients (*n* = 118), RDs (*n* = 60), and HDs (*n* = 36) using 193 immune parameters identified by high-dimensional flow cytometry (tables S5 and S6). COVID-19 patients were clearly separated from RDs and HDs in PCA space, whereas RDs and HDs largely overlapped (Fig. 2A). We investigated the immune features that drive this COVID-19 immune signature. Given the role of CD8 T cells in response to viral infection, we focused on this cell type. Six major CD8 T cell populations were examined by using the combination of CD45RA, CD27, CCR7, and CD95 cell surface markers to define naïve (CD45RA^+^CD27^+^CCR7^+^CD95^−^), central memory [CD45RA^−^CD27^+^CCR7^+^ (CM)], effector memory [CD45RA^−^CD27^+^CCR7^−^ (EM1), CD45RA^−^CD27^−^CCR7^+^ (EM2), CD45RA^−^CD27^−^CCR7^−^ (EM3)], and EMRA (CD45RA^+^CD27^−^CCR7^−^) (Fig. 2B) CD8 T cells. Among the CD8 T cell populations, there was an increase in the EM2 and EMRA populations and a decrease in EM1 (Fig. 2C). Furthermore, the frequency of CD39^+^ cells was increased in COVID-19 patients compared with HDs (Fig. 2D). Although the frequency of PD-1^+^ cells was not different in the total CD8 population (Fig. 2D), it was increased for both CM and EM1 (fig. S2A). Finally, all major CD8 T cell naïve and memory populations in RDs were comparable to those in HDs (Fig. 2, C and D, and fig. S2A).

Most acute viral infections induce proliferation and activation of CD8 T cells detectable by increases in KI67 or coexpression of CD38 and HLA-DR (*34*, *35*). There was a significant increase in KI67^+^ and also HLA-DR^+^CD38^+^ non-naïve CD8 T cells in COVID-19 patients relative to HDs or RDs (Fig. 2, E and F). In COVID-19 patients compared with HDs and RDs, KI67^+^ CD8 T cells were increased across all subsets of non-naïve CD8 T cells, including CM and EM1 populations (fig. S2B). These data indicate broad T cell activation, potentially driven by bystander activation and/or homeostatic proliferation in addition to antigen-driven activation of virus-specific CD8 T cells. This activation phenotype was confirmed by HLA-DR and CD38 coexpression that was significantly increased for all non-naïve CD8 T cell subsets (Fig. 2F and fig. S2C). However, the magnitude of the KI67^+^ or CD38^+^HLA-DR^+^ CD8 T cells varied widely in this cohort. The frequency of KI67^+^ CD8 T cells correlated with the frequency of CD38^+^HLA-DR^+^ CD8 T cells (fig. S2D). However, the frequency of CD38^+^HLA-DR^+^ T cells, but not KI67^+^ CD8 T cells, was elevated in COVID-19 patients who had concomitant infection with another microbe but was not affected by preexisting immunosuppression or treatment with steroids (fig. S2E). Moreover, these changes in CD8 T cell subsets in COVID-19 patients did not show clear correlations with individual metrics of clinical disease such as hsCRP or D-dimer (fig. S2E), although the frequency of KI67^+^ CD8 T cells was associated with elevated IL-6 and ferritin levels. Although CD8 T cell activation was common, ~20% of patients had no increase in KI67^+^ or CD38^+^HLA-DR^+^ CD8 T cells above the level found in HDs (Fig. 2, E and F). Thus, although robust CD8 T cell activation was a clear characteristic of many hospitalized COVID-19 patients, a substantial fraction of patients had little evidence of CD8 T cell activation in the blood compared with controls.

To gain more insights, we applied global high-dimensional mapping of the 27-parameter flow cytometry data. A t-distributed stochastic neighbor embedding (tSNE) representation of the data highlighted key regions of non-naïve CD8 T cells found preferentially in COVID-19 patients (Fig. 2G). A major region of this tSNE map present in COVID-19 patients, but not HDs or RDs, encompasses CD8 T cells enriched for expression of CD38, HLA-DR, KI67, CD39, and PD-1 (Fig. 2G), highlighting the coexpression of these activation markers with other features, including CD95 (i.e., FAS). Notably, although non-naïve CD8 T cells from RDs were highly similar to those from HDs, subtle differences existed, including in the lower region highlighted by T-bet and CX3CR1 (Fig. 2G). To further define and quantify these differences between COVID-19 patients and controls, we performed FlowSOM clustering (Fig. 2H) and compared expression of 14 CD8 T cell markers to identify each cluster (Fig. 2I). This approach identified an increase in cells in several clusters, including clusters 1, 2, and 5 in COVID-19 patients, reflecting CD45RA^+^CD27^−^CCR7^−^ EMRA-like populations that expressed CX3CR1 and varying levels of T-bet (Fig. 2, I and J) (“EMRA” denotes a subset of effector memory T cells reexpressing CD45RA). Clusters 12 and 14 contained CD27^+^HLA-DR^+^CD38^+^KI67^+^PD-1^+^ activated, proliferating cells and were more prevalent in COVID-19 patients (Fig. 2, I and J, and fig. S2F). By contrast, the central Eomes^+^CD45RA^−^CD27^+^CCR7^−^ EM1-like cluster 6 and T-bet^hi^CX3CR1^+^ cluster 11 were decreased in COVID-19 patients compared with HDs (Fig. 2, I and J, and fig. S2F). Thus, CD8 T cell responses in COVID-19 patients were characterized by populations of activated, proliferating CD8 T cells in a subgroup of patients.

## SARS-CoV-2 infection is associated with heterogeneous CD4 T cell responses and activation of CD4 T cell subsets

**Fig. 3:**
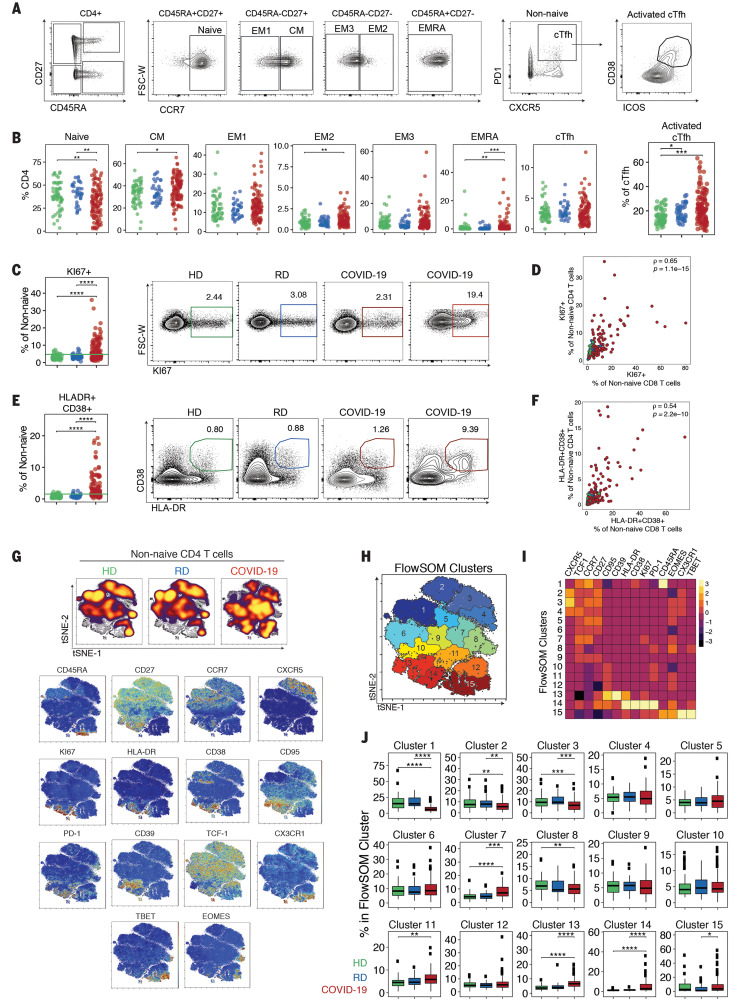
CD4 T cell activation in a subset of COVID-19 patients is associated with distinct CD4 T cell subsets. (**A**) Representative flow cytometry plots of the gating strategy for CD4 T cell subsets. (**B**) Frequencies of CD4 T cell subsets, as indicated. (**C**) Frequencies of KI67^+^ cells. The green line in the left panel denotes the upper decile of HDs. Representative flow cytometry plots are shown at right. (**D**) KI67^+^ cells from non-naïve CD4 T cells versus non-naïve CD8 T cells. Spearman correlation of COVID-19 patients is shown. (**E**) Frequencies of HLA-DR^+^CD38^+^ cells. The green line in the left panel denotes the upper decile of HDs. Representative flow cytometry plots are shown at right. (**F**) HLA-DR^+^CD38^+^ cells from non-naïve CD4 versus non-naïve CD8 T cells, Spearman correlation of COVID-19 patients is shown. (**G**) (Top) Global viSNE projection of non-naïve CD4 T cells for all participants pooled, with non-naïve CD4 T cells from HDs, RDs, and COVID-19 patients concatenated and overlaid. (Bottom) viSNE projections of indicated protein expression. (**H**) viSNE projection of non-naïve CD4 T cell clusters identified by FlowSOM clustering. (**I**) MFI as indicated (column-scaled *z*-scores). (**J**) Percentage of non-naïve CD4 T cells from each cohort in each FlowSOM cluster. Boxes represent IQRs. (B, C, E, and J) Each dot represents an individual HDs (green), RDs (blue), or COVID-19 patient (red). Significance was determined by unpaired Wilcoxon test with BH correction: *P < 0.05, **P < 0.01, ***P < 0.001, and ****P < 0.0001.

We next examined six well-defined CD4 T cell subsets as above for the CD8 T cells, including naïve; EM1, -2, and -3; CM; and EMRA (Fig. 3A). Given the potential role of antibodies in the response to SARS-CoV-2 (*27*, *29*), we also analyzed circulating T follicular helper (T_FH_) cells [CD45RA^−^PD-1^+^CXCR5^+^ (cT_FH_) (*36*)] and activated circulating T_FH_ cells [CD38^+^ICOS^+^ (activated cT_FH_)], the latter of which may be more reflective of recent antigen encounter and emigration from the germinal center (*37*, *38*) (Fig. 3A). These analyses revealed a relative loss of naïve CD4 T cells compared with controls, but increased EM2 and EMRA (Fig. 3B). The frequency of activated but not total cT_FH_ cells was statistically increased in COVID-19 patients compared with HDs, though this effect appeared to be driven by a subgroup of patients (Fig. 3B). Notably, activated cT_FH_ frequencies were also higher in RDs than in HDs (Fig. 3B), perhaps reflecting residual COVID-19 responses in that group. Frequencies of KI67^+^ or CD38^+^HLA-DR^+^ non-naïve CD4 T cells were increased in COVID-19 patients (Fig. 3, C and E); however, this change was not equivalent across all CD4 T cell subsets. The most substantial increases in both KI67^+^ and CD38^+^HLA-DR^+^ cells were found in the effector memory populations (EM1, EM2, EM3) and in cT_FH_ cells (fig. S3, A and B). Although some individuals had increased activation of EMRA, this response was less pronounced. By contrast, PD-1 expression was increased in all other non-naïve populations compared with HDs or RDs (fig. S3C). Coexpression of CD38 and HLA-DR by non-naïve CD4 T cells correlated with the frequency of KI67^+^ non-naïve CD4 T cells (fig. S3D). Moreover, the frequency of total non-naïve CD4 T cells that were CD38^+^HLA-DR^+^ correlated with the frequency of activated cT_FH_ cells (fig. S3E). In general, the activation of CD4 T cells was correlated with the activation of CD8 T cells (Fig. 3, D and F). However, whereas about two-thirds of COVID-19 patients had KI67^+^ non-naïve CD4 or CD8 T cell frequencies above controls, about one-third had no increase in frequency of KI67^+^ CD4 or CD8 T cells above that observed in HDs (Fig. 3, D and F). Moreover, although most patients had similar proportions of activated CD4 and CD8 T cells, a subgroup of patients had disproportionate activation of CD4 T cells relative to CD8 T cells (Fig. 3, D and F). KI67^+^ and CD38^+^HLA-DR^+^ non-naïve CD4 T cell frequencies correlated with ferritin and with APACHE III score (fig. S3F), suggesting a relationship between CD4 T cell activation and disease severity. Immunosuppression did not affect CD4 T cell activation; however, early steroid administration was weakly associated with CD4 T cell KI67 expression (fig. S3F). Together, these data indicate that T cell activation in COVID-19 patients is similar to what has been observed in other acute infections or vaccinations (*37*, *39*, *40*) and identify patients with high, low, or essentially no T cell response on the basis of KI67^+^ or CD38^+^HLA-DR^+^ expression compared with control individuals.

Projecting the global CD4 T cell differentiation patterns into the high-dimensional tSNE space again identified major alterations in the CD4 T cell response in COVID-19 patients compared with HDs and RDs (Fig. 3G). In COVID-19 infection, there was a notable increase in density in tSNE regions that mapped to expression of CD38, HLA-DR, PD1, CD39, KI67, and CD95 (Fig. 3G), similar to CD8 T cells. To gain more insight into these CD4 T cell changes, we again used a FlowSOM clustering approach (Fig. 3, H and I). This analysis identified an increase in clusters 13 and 14 (representing populations that express HLA-DR, CD38, PD1, KI67 and CD95) as well as cluster 15 (containing Tbet^+^CX3CR1^+^ effector-like CD4 T cells) in COVID-19 patients compared with HDs and RDs (Fig. 3, I and J, and fig. S3G). By contrast, this clustering approach identified reduction in CXCR5^+^ cT_FH_-like cells (clusters 2 and 3) in COVID-19 participants compared with HDs (Fig. 3, I and H). Collectively, the results of this multidimensional analysis reveal distinct populations of activated and proliferating CD4 T cells that were enriched in COVID-19 patients.

A key feature of COVID-19 is thought to be an inflammatory response that, at least in some patients, is linked to clinical disease manifestation (*2*, *4*) and high levels of chemokines and cytokines, including IL-1RA, IL-6, IL-8, IL-10, and CXCL10 (*11*, *41*). To investigate the potential connection of inflammatory pathways to T cell responses, we performed 31-plex Luminex analysis on paired plasma and culture supernatants of αCD3- and αCD28-stimulated PBMCs from a subset of COVID-19 patients and HD controls. Owing to biosafety restrictions, we were able to study only eight COVID-19 patient blood samples that were confirmed negative for SARS-CoV-2 RNA by polymerase chain reaction (PCR) (fig. S4A). Half of these COVID-19 patients had plasma CXCL10 concentrations that were ~15 times as high as those of HD controls, whereas the remainder showed only a limited increase (fig. S4B). CXCL9, CCL2, and IL-1RA were also significantly increased. By contrast, chemokines involved in the recruitment of eosinophils (eotaxin) or activated T cells (CCL5) were decreased. IL-6 was not elevated in this group of patients, in contrast to the subset of individuals tested clinically (fig. S1B), potentially because IL-6 was measured in the hospital setting, often when systemic inflammation was suspected. After stimulation in vitro, PBMCs from COVID-19 patients produced more CCL2, CXCL10, eotaxin, and IL-1RA than those from HDs (fig. S4, C and D), and concentrations of CXCL10 and CCL2 correlated between the matched supernatant from stimulated PBMCs and plasma samples (fig. S4E). Finally, we investigated whether CD8 T cells from COVID-19 patients were capable of producing interferon-γ (IFNγ) after polyclonal stimulation. After stimulation with αCD3 and αCD28, similar proportions of CD8 T cells from COVID-19 patients and HD controls produced IFNγ, which suggests that PBMCs from COVID-19 patients were responsive to T cell receptor cross-linking (fig. S4, F to H). The ability of T cells to produce IFNγ after stimulation occurred in patients with increases in KI67 as well as patients with low KI67 (fig. S4, F to H). Taken together, these data support the notion that a subgroup of COVID-19 patients has elevated systemic cytokines and chemokines, including myeloid-recruiting chemokines.

## COVID-19 infection is associated with increased frequencies of PBs and proliferation of memory B cell subsets

**Fig. 4:**
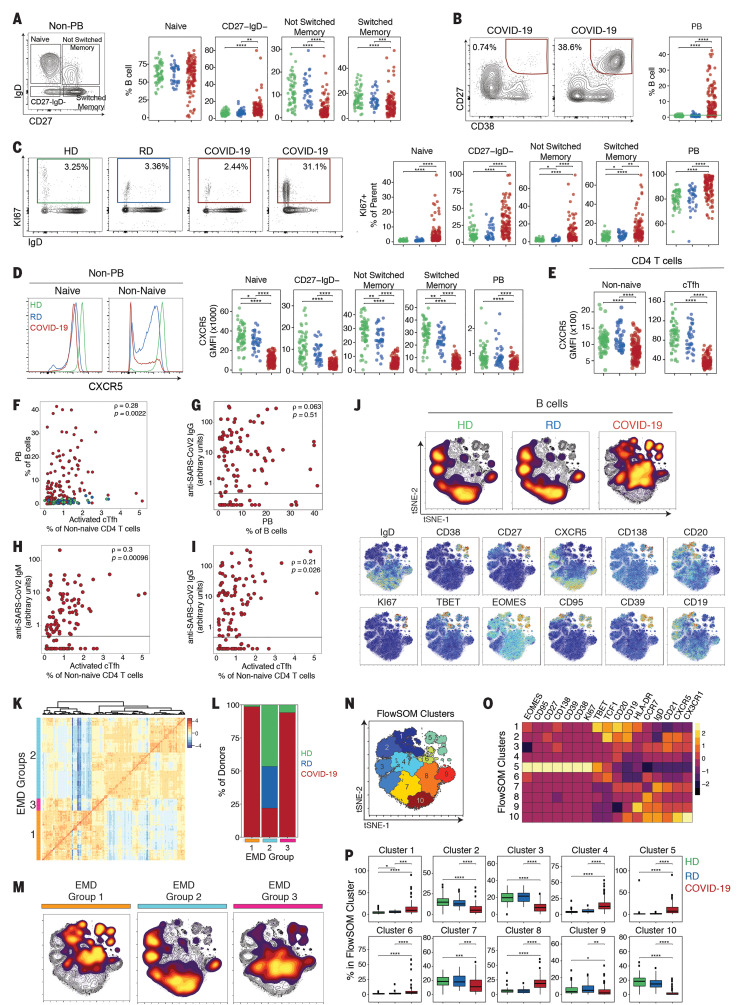
Deep profiling of COVID-19 patient B cell populations reveals robust PB populations and other B cell alterations. (**A**) Gating strategy and frequencies of non-PB B cell subsets. (**B**) Representative flow cytometry plots and frequencies of PBs. The green line in the right panel denotes the upper decile of HDs. (**C**) Representative flow cytometry plots and frequencies of KI67^+^ B cells. (**D**) (Left) Representative histograms of CXCR5 expression; (right) CXCR5 geometric MFI (GMFI) of B cell subsets. (**E**) CXCR5 GMFI of non-naïve CD4 T cells and cT_FH_ cells. (**F**) Spearman correlation between PBs and activated cT_FH_ cells. (**G**) Spearman correlation between PBs and anti–SARS-CoV-2 IgG. (**H** and **I**) Spearman correlation between activated cT_FH_ cells and anti–SARS-CoV-2 (H) IgM and (I) IgG. (**J**) (Top) Global viSNE projection of B cells for all participants pooled, with B cell populations of each cohort concatenated and overlaid. (Bottom) viSNE projections of expression of the indicated proteins. (**K**) Hierarchical clustering of EMD using Pearson correlation, calculated pairwise for B cell populations for all participants (row-scaled *z*-scores). (**L**) Percentage of cohort in each EMD group. (**M**) Global viSNE projection of B cells for all participants pooled, with EMD groups 1 to 3 concatenated and overlaid. (**N**) B cell clusters identified by FlowSOM clustering. (**O**) MFI as indicated (column-scaled *z*-scores). (**P**) Percentage of B cells from each cohort in each FlowSOM cluster. Boxes represent IQRs. (A to F and P) Dots represent individual HDs (green), RDs (blue), or COVID-19 (red) participants. (A to E and P) Significance was determined by unpaired Wilcoxon test with BH correction: *P < 0.05, **P < 0.01, ***P < 0.001, and ****P < 0.0001. (G to I) The black horizontal line represents the positive threshold.

B cell subpopulations were also altered in people with COVID-19. Whereas naïve B cell frequencies were similar in COVID-19 patients and RDs or HDs, the frequencies of class-switched (IgD^−^CD27^+^) and not–class-switched (IgD^+^CD27^+^) memory B cells were significantly reduced (Fig. 4A). Conversely, frequencies of CD27^−^IgD^−^ B cells and CD27^+^CD38^+^ PBs were often markedly increased (Fig. 4, A and B). In some cases, PBs represented >30% of circulating B cells, similar to levels observed in acute Ebola or dengue virus infections (*42*, *43*). However, these PB responses were observed in only about two-thirds of patients, with the remaining patients displaying PB frequencies similar to those in HDs and RDs (Fig. 4B). KI67 expression was markedly elevated in all B cell subpopulations in COVID-19 patients compared with either control group (Fig. 4C). This observation suggests a role for an antigen-driven response to infection- and/or lymphopenia-driven proliferation. Higher KI67 levels in PBs may reflect recent generation in COVID-19 patients relative to HDs or RDs. CXCR5 expression was also reduced on all major B cell subsets in COVID-19 patients (Fig. 4D). Loss of CXCR5 was not specific to B cells, however, as expression was also decreased on non-naïve CD4 T cells (Fig. 4E). Changes in the B cell subsets were not associated with coinfection, immune suppression, or treatment with steroids or other clinical features, though a possible negative association of IL-6 and PBs was revealed (fig. S5A). These observations suggest that the B cell response phenotype of COVID-19 was not simply due to systemic inflammation.

During acute viral infections or vaccination, PB responses are transiently detectable in the blood and correlate with cT_FH_ responses (*40*). Comparing the frequency of PBs to the frequency of total cT_FH_ cells or activated cT_FH_ cells, however, revealed a weak correlation only with activated cT_FH_ cells (Fig. 4F and fig. S5, B and C). Furthermore, some patients had robust activated cT_FH_ responses but PB frequencies similar to those of controls, whereas other patients with robust PB responses had relatively low frequencies of activated cT_FH_ cells (Fig. 4F and fig. S5, B and C). There was also an association between PB frequency and CD38^+^HLA-DR^+^ or KI67^+^ CD4 T cells that might reflect a role for non-CXCR5^+^ CD4 T cell help (fig. S5D), but such a relationship did not exist for the equivalent CD8 T cell populations (fig. S5E). Although ~70% of the COVID-19 patients analyzed in our study made antibodies against SARS-CoV-2 spike protein [79 of 111 IgG; 77 of 115 IgM (*44*)], antibody levels did not correlate with PB frequencies (Fig. 4G and fig. S5F). The occasional lack of antibody did not appear to be related to immunosuppression in a small number of patients (fig. S5G). The lack of PB correlation with antibody suggests that a proportion of these large PB responses were: (i) generated against SARS-CoV-2 antigens other than the spike protein or (ii) inflammation driven and perhaps nonspecific or low affinity. Notably, anti–SARS-CoV-2 IgG and IgM levels correlated with the activated, but not total, cT_FH_ response, which suggests that at least a proportion of cT_FH_ cells were providing SARS-CoV-2–specific help to B cells (Fig. 4, H and I, and fig. S5, H and I). Although defining the precise specificity of the robust PB populations will require future studies, these data suggest that at least some of the PB response is specific for SARS-CoV-2.

Projecting the flow cytometry data for B cells from HDs, RDs, and COVID-19 patients in tSNE space revealed a distinct picture of B cell populations in COVID-19 patients compared with controls, whereas populations in RDs and HDs were similar (Fig. 4J and fig. S5J). The COVID-19 patient B cell phenotype was dominated by the loss of CXCR5 and IgD compared with B cells from HDs and RDs (Fig. 4J). Moreover, the robust PB response was apparent in the upper right section, highlighted by CD27, CD38, CD138, and KI67 (Fig. 4J). The expression of KI67 and CD95 in these CD27^+^CD38^+^CD138^+^ PBs (Fig. 4J) may suggest recent generation and/or emigration from germinal centers. We next asked whether there were different groups of COVID-19 patients (or HDs and RDs) with global differences in the B cell response. We used the Earth mover’s distance (EMD) metric (*45*) to calculate similarities between the probability distributions within the tSNE map (Fig. 4J) and clustered data so that individuals with the most-similar distributions grouped together (Fig. 4K). The majority of COVID-19 patients fell into two distinct groups (EMD groups 1 and 3; Fig. 4L), suggesting two major immunotypes of the B cell response. The remainder of the COVID-19 patients (~25%) clustered with the majority of the HD and all of the RD controls, supporting the observation that some individuals had limited evidence of response to infection in their B cell compartment. To identify the population differences between HDs, RDs, and COVID-19 patients, we performed FlowSOM clustering on the tSNE map and overlaid each individual EMD group onto this same tSNE map (Fig. 4, M and N). EMD group 2, containing mostly HDs and RDs, was enriched for naïve B cells (IgD^+^CD27^−^, cluster 10) and CXCR5^+^IgD^−^CD27^+^ switched memory cells (cluster 2), and indeed, clusters 2 and 10 were statistically reduced in COVID-19 patients (Fig. 4P). EMD groups 1 and 3 displayed distinct patterns across the FlowSOM clusters. B cells from individuals in EMD group 1 were enriched for FlowSOM clusters 1, 5, and 6, all of which were increased in COVID-19 patients (Fig. 4P). FlowSOM clusters 1 and 6 captured T-bet^+^ memory B cells, whereas FlowSOM cluster 5 contained the CD27^+^CD38^+^CD138^+^KI67^+^ PBs, all of which were enriched in COVID-19 patients relative to controls (Fig. 4, O and P, and fig. S5K). By contrast, B cells from COVID-19 patients in EMD group 3 also showed enrichment for the PB FlowSOM cluster 5, though less prominent than for EMD group 1, but the T-bet^+^ memory B cell cluster 1 was substantially reduced in EMD group 3. Thus, B cell responses—most often characterized by elevated PBs, decreases in memory B cell subsets, enrichment in a T-bet^+^ B cell subset, and loss of CXCR5 expression—were evident in many hospitalized COVID-19 patients. Whether all of these changes in the B cell compartment were due to direct antiviral responses is unclear. Although there was heterogeneity in the B cell responses, COVID-19 patients fell into two distinct patterns containing activated B cell responses and a third group of patients with little evidence of an active B cell response.

## Temporal changes in immune cell populations occur during COVID-19

**Fig. 5:**
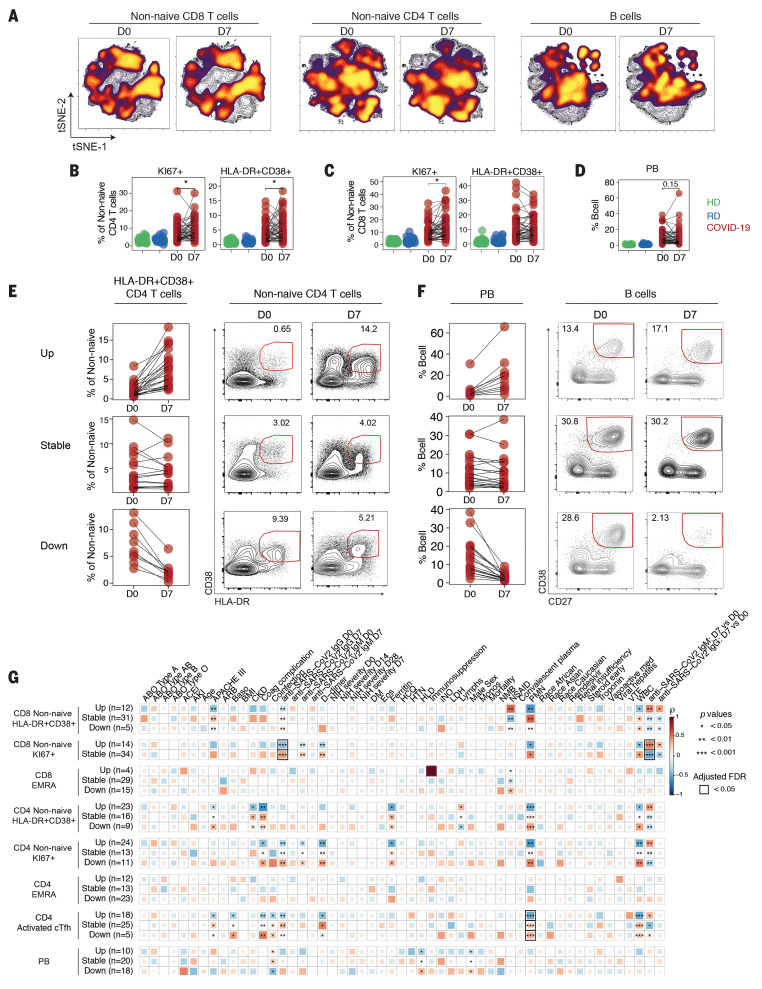
Temporal relationships between immune responses and disease manifestation. (**A**) Global viSNE projection of non-naïve CD8 T cells, non-naïve CD4 T cells, and B cells for all participants pooled, with cells from COVID-19 patients at D0 and D7 concatenated and overlaid. Frequencies of (**B**) KI67^+^ and HLA-DR^+^CD38^+^ CD4 T cells, (**C**) KI67^+^ and HLA-DR^+^CD38^+^ CD8 T cells, or (**D**) PBs as indicated for HDs (green), RDs (blue), or COVID-19 patients (red), with paired samples at D0 and D7 indicated by connecting lines. Significance was determined by paired Wilcoxon test: *P < 0.05, **P < 0.01, ***P < 0.001, and ****P < 0.0001. Longitudinal patterns (see Materials and methods) of (**E**) HLA-DR^+^CD38^+^ CD4 T cells or (**F**) PBs in COVID-19 patients shown as frequency and representative flow cytometry plots. (**G**) Spearman correlations of clinical parameters with longitudinal fold changes in immune populations.

A key question for hospitalized COVID-19 patients is how immune responses change over time. Thus, we used the global tSNE projections of overall CD8 T cell, CD4 T cell, and B cell differentiation states to investigate temporal changes in these populations between D0 and D7 of hospitalization (Fig. 5A). Combining data for all patients revealed considerable stability of the tSNE distributions between D0 and D7 in CD8 T cell, CD4 T cell, and B cell populations, particularly for the key regions of interest discussed above. For example, for CD8 T cells, the region of the tSNE map containing KI67^+^ and CD38^+^HLA-DR^+^ CD8 T cell populations that was enriched in COVID-19 patients at D0 (Fig. 2) was preserved at D7 (Fig. 5A). A similar temporal stability of CD4 T cell and B cell activation was also observed (Fig. 5A).

Given this apparent stability between D0 and D7, we next investigated temporal changes in lymphocyte subpopulations of interest. Although there were no obvious temporal changes in major phenotypically defined CD4 and CD8 T cell or B cell subsets, including PBs (Fig. 5D), the frequencies of HLA-DR^+^CD38^+^ and KI67^+^ non-naïve CD4 (Fig. 5B) and KI67^+^ non-naïve CD8 T cells were statistically increased at D7 compared with D0 (Fig. 5C).

However, in all cases, these temporal patterns were complex, with frequencies of subpopulations in individual patients appearing to increase, decrease, or stay the same over time. To quantify these interpatient changes, we used a previously described dataset (*46*) to define the stability of populations of interest in healthy individuals over time. We then used the range of this variation over time to identify COVID-19 patients with changes in immune cell subpopulations beyond that expected in healthy people (see Materials and methods section). With this approach, ~50% of patients had an increase in HLA-DR^+^CD38^+^ non-naïve CD4 T cells over time, whereas these cells were stable in ~30% of patients and decreased in the remaining ~20% (Fig. 5E). For KI67^+^ non-naïve CD8 T cells, there were no individuals in whom the response decreased. Instead, this proliferative CD8 T cell response stayed stable (~70%) or increased (~30%) (fig. S6A). Notably, for patients in the stable category, the median frequency of KI67^+^ non-naïve CD8 T cells was ~10%, almost 10 times as high as the ~1% detected for HDs and RDs (Figs. 5C and 2E), suggesting a sustained CD8 T cell proliferative response to infection. A similar pattern was observed for HLA-DR^+^CD38^+^ non-naïve CD8 cells (fig. S6B): Only ~10% of patients had a decrease in this population, whereas ~65% were stable and ~25% had an increase over time. The high and even increasing activated or proliferating CD8 and CD4 T cell responses over ~1 week during acute viral infection contrasted with the sharp peak of KI67 in CD8 and CD4 T cells during acute viral infections, including smallpox vaccination with live vaccinia virus (*47*), live attenuated yellow fever vaccine YFV-17D (*48*), acute influenza virus infection (*49*), and acute HIV infection (*35*). Approximately 42% of patients had sustained PB responses, at high levels (>10% of B cells) in many cases (Fig. 5F). Thus, some patients displayed dynamic changes in T cell or B cell activation over 1 week in the hospital, but other patients remained stable. In the latter case, some patients remained stable without clear activation of key immune populations, whereas others had stable T and/or B cell activation or numerical perturbation (fig. S6C).

We next asked whether these T and B cell dynamics are related to clinical measures of COVID-19. To do this, we correlated changes in immune features from D0 to D7 with clinical information (Fig. 5G). These analyses revealed distinctive correlations. Decreases in all populations of responding CD4 and CD8 T cells (HLA-DR^+^CD38^+^, KI67^+^, and activated cT_FH_) between D0 and D7 were positively correlated with PMN and WBC counts, suggesting a relationship between T cell activation and lymphopenia. Furthermore, decreases in CD4 and CD8 HLA-DR^+^CD38^+^ T cells positively correlated with APACHE III score. However, stable HLA-DR^+^CD38^+^ CD4 T cell responses correlated with coagulation complications and ferritin levels. Whereas decreasing activated cT_FH_ cells over time was related to coinfection, the opposite pattern was observed for PBs. Increases in proliferating KI67^+^ CD4 and CD8 T cells over time were positively correlated to increasing anti–SARS-CoV-2 antibody from D0 to D7, suggesting that some individuals might have been hospitalized during the expansion phase of the antiviral immune response (Fig. 5G). Finally, neither remdesivir nor HCQ treatment correlated with any of these immune features (Fig. 5G). When we examined categorical rather than continuous clinical data, we found that 80% of patients with decreasing PBs over time had hyperlipidemia, whereas only 20% of patients with increasing PBs over time had this comorbidity (fig. S6D). All patients who had decreasing CD38^+^HLA-DR^+^ CD8 T cells from D0 to D7 were treated with early vasoactive medication or inhaled nitric oxide, whereas these treatments were less common for patients with stable or increasing CD38^+^HLA-DR^+^ CD8 T cells (fig. S6E). By contrast, vasoactive medication, inhaled nitric oxide, and early steroid treatment were equally common in patients with increasing or decreasing PBs (fig. S6D). Similar patterns were apparent for other T cell populations and these categorical clinical data (fig. S6F). Thus, the trajectory of change in the T and B cell response in COVID-19 patients was strongly connected to clinical metrics of disease.

## Identifying immunotypes and relationships between circulating B and T cell responses with disease severity in COVID-19 patients

**Fig. 6:**
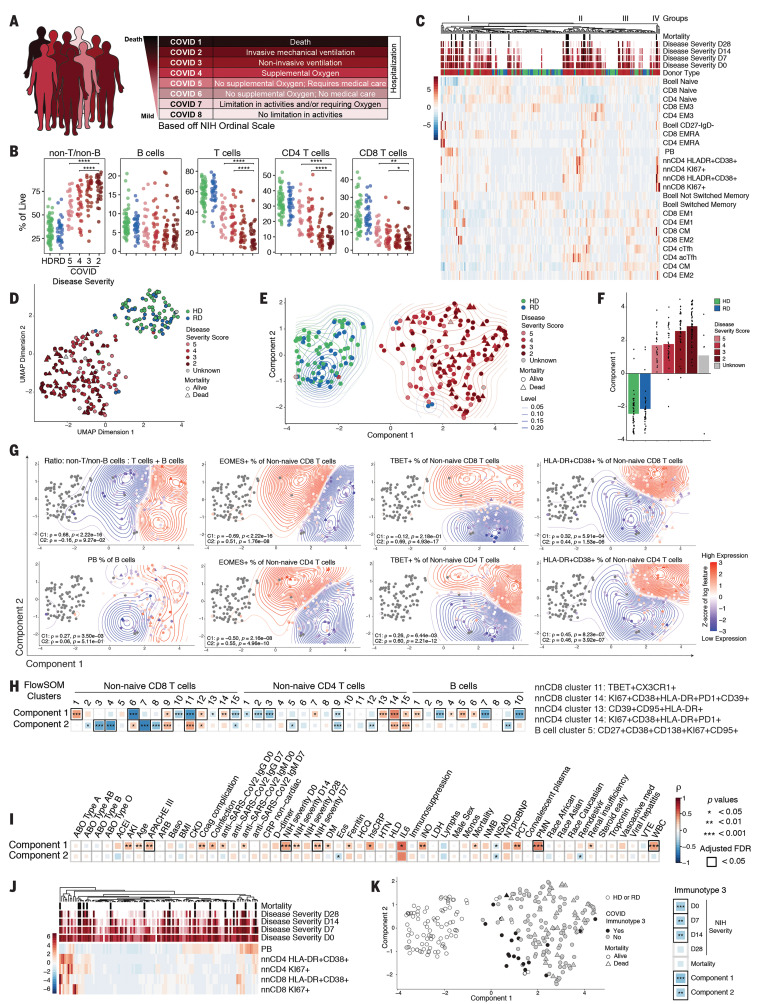
High-dimensional analysis of immune phenotypes with clinical data reveals distinct COVID-19 patient immunotypes. (**A**) NIH ordinal scale for COVID-19 clinical severity. (**B**) Frequencies of major immune subsets. Significance was determined by unpaired Wilcoxon test with BH correction: *P < 0.05, **P < 0.01, ***P < 0.001, and ****P < 0.0001. (**C**) Heatmap of indicated immune parameters by row; donor type, disease severity, and mortality are indicated across the top. (**D**) UMAP projection of aggregated flow cytometry data. (**E**) Transformed UMAP projection. Density contours were drawn separately for HDs, RDs, and COVID-19 patients (see Materials and methods). (**F**) Bars represent mean of UMAP component 1. Dots represent individual participants; bars are color-coded by participant group and/or severity score. (**G**) Density contour plots indicating variation of specified immune features across UMAP component coordinates. Relative expression (according to heat scale) is shown for both individual patients (points) and overall density (contours). Spearman’s rank correlation coefficient (ρ) and *P* value for each feature versus component 1 (C1) and component 2 (C2) are shown. (**H**) (Left) Spearman correlation between UMAP components 1 and 2 and FlowSOM clusters. (Right) Select FlowSOM clusters and their protein expression. (**I**) Spearman correlation between UMAP components 1 and 2 and clinical metadata. (**J**) Heatmap of immune parameters used to define immunotype 3 indicated by row; disease severity and mortality are indicated across the top. (**K**) (Left) Transformed UMAP projection; patient status for immunotype 3 indicated by color. (Right) Spearman correlation between immunotype 3 and disease severity, mortality, and UMAP components.

To further investigate the relationship between immune responses and COVID-19 trajectory, we stratified the COVID-19 patients (*n* = 125) into eight different categories, according to the NIH Ordinal Severity Scale, ranging from COVID 1 (death) and COVID 2 (requiring maximal clinical intervention) to COVID 8 (at home with no required care) (Fig. 6A). We then asked how changes in T and B cell populations defined above on D0 were related to disease severity. More severe disease was associated with lower frequencies of CD8 and CD4 T cells, with a greater effect on CD8 T cells in less severe disease (Fig. 6B). Taking all patients together, there were no statistically significant changes in the major T cell and B cell subsets related to disease severity, though some trends were present (fig. S7, A to C). By contrast, HLA-DR^+^CD38^+^ CD8 T cells as well as both KI67^+^ and HLA-DR^+^CD38^+^ CD4 T cells were increased in patients with more severe disease (fig. S7, D and E).

There were two challenges with extracting meaning from these data. First, there was considerable interpatient heterogeneity for each of these immune features related to disease severity score. Second, these binary comparisons (e.g., one immune subset versus one clinical feature) do not make full use of the high-dimensional information in this dataset. Thus, we next visualized major T and B cell subpopulation data as related to clinical disease severity score (Fig. 6C). Data were clustered according to immune features and then overlaid with the disease severity score over time for each patient. This analysis revealed groups of patients with similar composite immune signatures of T and B cell populations (Fig. 6C). When individual CD8 T cell, CD4 T cell, or B cell populations were examined, a similar concept of patient subgroups emerged (fig. S7, F, G, and H). These data suggested the idea of immunotypes of COVID-19 patients on the basis of integrated responses of T and B cells, though some individual cell types and/or phenotypes separated patients more clearly than others.

These approaches provided insight into potential immune phenotypes associated with patients with severe disease but were hindered by the small number of manually selected T or B cell subsets or phenotypes. We therefore next employed uniform manifold approximation and projection (UMAP) to distill the ~200 flow cytometry features (tables S5 and S6) representing the immune landscape of COVID-19 in two-dimensional space, creating compact meta-features (or components) that could then be correlated with clinical outcomes. This analysis revealed a clear trajectory from HDs to COVID-19 patients (Fig. 6D), which we centered and aligned with the horizontal axis (component 1) to facilitate downstream analysis (Fig. 6E). An orthogonal vertical axis coordinate (component 2) captured nonoverlapping aspects of the immune landscape. We next calculated the mean of component 1 for each patient group, with COVID-19 patients separated by severity score (Fig. 6E). The contribution of component 1 clearly increased in a stepwise manner with increasing disease severity (Fig. 6F). Notably, RDs were subtly positioned between HDs and COVID-19 patients. Component 1 remained an independent predictor of disease severity (*P* = 5.5 × 10^−5^) even after adjusting for the confounding demographic factors of age, sex, and race.

We next investigated how the UMAP components were associated with individual immune features (tables S5 and S6). UMAP component 1 captured immune features, including the relative loss of CD4 and CD8 T cells and increase in the ratio of non-B and non-T cells to T and B cells (Fig. 6G). PBs also associated with component 1 (Fig. 6G). Other individual B cell features were differentially captured by UMAP components 1 and 2. Component 1 contained a signal for T-bet^+^ PB populations (table S5), whereas component 2 was enriched for T-bet^+^ memory B cells and CD138^+^ PB populations (table S6). Activated HLA-DR^+^CD38^+^ and KI67^+^ CD4 and CD8 T cells had contributions to both components, with these features residing in the upper right corner of the UMAP plot (Fig. 6, G and H, and fig. S8, A to D). By contrast, T-bet^+^ non-naïve CD8 T cells were strongly associated with component 2, whereas T-bet^+^ non-naïve CD4 T cells were linked to component 1 (Fig. 6G and tables S5 and S6). Eomes^+^ CD8 or CD4 T cells were both associated with component 2 and negatively associated with component 1 (Fig. 6G and tables S5 and S6).

We next took advantage of the FlowSOM clustering in Figs. 2 to 4 that identified individual immune cell types most perturbed in COVID-19 patients and linked these FlowSOM clusters to UMAP components (Fig. 6H). For non-naïve CD8 T cells, FlowSOM cluster 11, which contained T-bet^+^CX3CR1^+^ but nonproliferating effector-like cells, was positively correlated with UMAP component 2 and negatively correlated with component 1 (Fig. 6H). By contrast, FlowSOM cluster 14, which contained activated, proliferating PD-1^+^CD39^+^ cells that might reflect either recently generated effector or exhausted CD8 T cells (*50*), was strongly associated with UMAP component 1 (Fig. 6H). For CD4 T cells, FlowSOM cluster 14, containing activated, proliferating CD4 T cells, was captured by both UMAP components, whereas a second activated CD4 T cell population that also expressed CD95 (FlowSOM cluster 13) was captured by only UMAP component 1 (Fig. 6H). In addition, component 1 was negatively correlated with CD4 T cell FlowSOM clusters 2 and 3 that contained cT_FH_ cells (Fig. 6H). Finally, for B cells, the FlowSOM cluster of T-bet^+^CD138^+^ PBs (cluster 5) was positively correlated with component 1, whereas the T-bet^−^CD138^+^ cluster 3 was negatively correlated with this same component (Fig. 6H). Locations in the UMAP immune landscape were dynamic, changing from D0 to D7 for both components, consistent with the data in Fig. 5 and fig. S9, A to F. The most dynamic changes in component 1 were associated with the largest increases in IgM antibody levels (fig. S9G).

Given the association of UMAP component 1 with disease severity, we next examined the connections between UMAP components and individual clinical features. UMAP component 1 correlated with several clinical measurements of inflammation (e.g., ferritin, hsCRP, IL-6), coinfection, organ failure (APACHE III), and acute kidney disease and renal insufficiency (Fig. 6I). However, although D-dimer level was elevated, this feature did not correlate with UMAP component 1, whereas coagulation complication did (Fig. 6I). Several antibody features also correlated with component 1, consistent with some of the immune features discussed above. By contrast, component 2 lacked positive correlation to many of these clinical features of disease and was negatively correlated to eosinophil count, nonsteroidal anti-inflammatory drug (NSAID) use, and subsequent treatment with remdesivir (Fig. 6I). UMAP component 1, not component 2, also correlated with mortality, although there were clearly patients with high component 2 but low component 1 who died from COVID-19 (Fig. 6E). These data indicate that the immune features captured by UMAP component 1 have a strong relationship to many features of disease severity, whereas other features of immune dynamics during COVID-19 captured by UMAP component 2 have a distinct relationship with clinical disease presentation.

More-positive values in UMAP components 1 or 2 captured mainly signals of change or differences in individual immune features in COVID-19 patients compared with HDs and RDs. UMAP component 1 captured an immunotype (immunotype 1) characterized by effector or highly activated CD4 T cells, low cT_FH_ cells, some CD8 EMRA-like activation, possibly hyperactivated CD8 T cells, and Tbet^+^ PBs, whereas component 2 (immunotype 2) captured Tbet^bright^ effector-like CD8 T cells, had less robust CD4 T cell activation, and had some features of proliferating B cells (Fig. 6G and fig. S8). However, the data presented in Figs. 1 to 5 also suggested a subset of patients with minimal activation of T and B cell responses. To investigate this immune signature, we identified 20 patients who had responses more similar to those of HDs and RDs for five activated or responding B and T cell populations (Fig. 6J, middle, and fig. S10). If UMAP components 1 and 2 captured two distinct immunotypes of patient responses to SARS-CoV-2 infection, this group of 20 patients represents a third immunotype. Immunotype 3 was negatively associated with UMAP components 1 and 2 and negatively associated with disease severity, which suggests that a less robust immune response during COVID-19 was associated with less severe pathology (Fig. 6K and fig. S10), despite the fact that these patients were hospitalized with COVID-19. These data further emphasize the different ways in which patients can present with and possibly die from COVID-19. These patterns may be related to preexisting conditions in combination with immune response characteristics. It is likely that additional immune features, such as comprehensive serum cytokine measurements, will improve this model. Nevertheless, the current computational approach integrating deep immune profiling with disease severity trajectory and other clinical information revealed distinct patient immunotypes linked to distinct clinical outcomes (fig. S11).

## Discussion

The T and B cell response to SARS-CoV-2 infection remains poorly understood. Some studies suggest that an overaggressive immune response leads to immunopathology (*51*), whereas others suggest that the mechanism is T cell exhaustion or dysfunction (*12*–*14*). Autopsies revealed high virus levels in the respiratory tract and other tissues (*52*), suggesting ineffective immune responses. Nevertheless, nonhospitalized individuals who recovered from COVID-19 had evidence of virus-specific T cell memory (*53*). SARS-CoV-2–specific antibodies are also found in convalescent individuals, and patients are currently being treated with convalescent plasma therapy (*30*, *54*). However, COVID-19 patients in intensive care units (ICUs) have SARS-CoV-2–specific antibodies (*30*), raising the question of why patients with these antibody responses are not controlling disease. In general, these previous studies have reported on single patients or small cohorts and thus do not achieve comprehensive deep immune profiling of larger numbers of hospitalized COVID-19 patients. Such knowledge would address the critical question of whether there is a common profile of immune dysfunction in critically ill patients. Such data would also help guide testing of therapeutics to enhance, inhibit, or otherwise tailor the immune response in COVID-19 patients.

To elucidate the immune response patterns of hospitalized patients with COVID-19, we studied a cohort of ~125 patients. We used high-dimensional flow cytometry to perform deep immune profiling of individual B and T cell populations, with temporal analysis of immune changes during infection, and combined this profiling with extensive clinical data to understand the relationships between immune responses to SARS-CoV-2 and disease severity. This approach led us to several key findings. First, a defining feature of COVID-19 in hospitalized patients is heterogeneity of the immune response. Many COVID-19 patients displayed robust CD8 T cell and/or CD4 T cell activation and proliferation and PB responses, though a substantial subgroup of patients (~20%) had minimal detectable responses compared with controls. Furthermore, even within those patients who mounted detectable B and T cell responses during COVID-19, the immune characteristics of the responses were heterogeneous. With the use of deep immune profiling, we identified three immunotypes in hospitalized COVID-19 patients: (i) robust activation and proliferation of CD4 T cells, relative lack of cT_FH_ cells, modest activation of EMRA-like cells, highly activated or exhausted CD8 T cells, and a signature of T-bet^+^ PBs (immunotype 1); (ii) Tbet^bright^ effector-like CD8 T cell responses, less robust CD4 T cell responses, and Ki67^+^ PBs and memory B cells (immunotype 2); and (iii) an immunotype largely lacking detectable lymphocyte response to infection, which suggests a failure of immune activation (immunotype 3). UMAP embedding further resolved the T cell–activation immunotype, suggesting a link between CD4 T cell activation, immunotype 1, and increased severity score. Although differences in age and race existed between the cohorts and could affect some immune variables, the major UMAP relationships were preserved even after correcting for these variables. Thus, these immunotypes may reflect fundamental differences in the ways in which patients respond to SARS-CoV-2 infection.

A second key observation from these studies was the robust PB response. Some patients had PB frequencies rivaling those found in acute Ebola or dengue infection (*34*, *42*, *43*, *55*). Furthermore, blood PB frequencies are typically correlated with blood-activated cT_FH_ responses (*40*). However, in COVID-19 patients, this relationship between PBs and activated cT_FH_ cells was weak. The lack of relationship between these two cell types in this disease could be due to T cell–independent B cell responses, lack of activated cT_FH_ cells in peripheral blood at the time point analyzed, or lower CXCR5 expression observed across lymphocyte populations, making it more difficult to identify cT_FH_ cells. Activated (CD38^+^HLA-DR^+^) CD4 T cells could play a role in providing B cell help, perhaps as part of an extrafollicular response, but such a connection was not robust in the current data. Most ICU patients made SARS-CoV-2–specific antibodies, suggesting that at least part of the PB response was antigen specific. Indeed, the cT_FH_ response did correlate with antibodies, which indicates that at least some of the humoral response is targeted against the virus. Future studies will be needed to address the antigen specificity, ontogeny, and role in pathogenesis for these robust PB responses.

A notable feature of some patients with strong T and B cell activation and proliferation was the durability of the PB response. This T and B cell activation was interesting considering the clinical lymphopenia in many patients. However, this lymphopenia was preferential for CD8 T cells. It may be notable that such focal lymphopenia preferentially affecting CD8 T cells is also a feature of acute Ebola infection of macaques and is associated with CD95 expression and severe disease (*55*). Indeed, CD95 was associated with activated T cell clusters in COVID-19. Nevertheless, the frequency of the KI67^+^ or CD38^+^HLA-DR^+^ CD8 and CD4 T cell responses in COVID-19 patients was similar in magnitude to those of other acute viral infections or live attenuated vaccines in humans (*47*–*49*). However, during many acute viral infections, the period for peak CD8 or CD4 T cell responses and the window for PB detection in peripheral blood are relatively short (*43*, *56*, *57*). The stability of CD8 and CD4 T cell activation and PB responses during COVID-19 suggests a prolonged period of peak immune responses at the time of hospitalization or perhaps a failure to appropriately down-regulate responses in some patients. These ideas would fit with an overaggressive immune response and/or “cytokine storm” (*2*) in this subset of patients. Indeed, in some patients, we found elevated serum cytokines and that stimulation of T cells in vitro provoked cytokines and chemokines capable of activating and recruiting myeloid cells. A key question will be how to identify these patients for selected immune-regulatory treatment while avoiding treating patients with already weak T and B cell responses.

An additional major finding was the ability to connect immune features to disease severity at the time of sampling as well as to the trajectory of disease severity change over time. Using correlative analyses, we observed relationships between features of the different immunotypes, patient comorbidities, and clinical features of COVID-19. By integrating ~200 immune features with extensive clinical data, disease severity scores, and temporal changes, we built an integrated computational model that connected patient immune response phenotype to disease severity. This UMAP embedding approach allowed us to connect these integrated immune signatures to specific clinically measurable features of disease. The integrated immune signatures captured by components 1 and 2 in this UMAP model provided support for the concept of immunotypes 1 and 2. These analyses suggested that immunotype 1—composed of robust CD4 T cell activation, paucity of cT_FH_ cells with proliferating effector or exhausted CD8 T cells, and T-bet^+^ PB involvement—was connected to more-severe disease, whereas immunotype 2—characterized by more traditional effector CD8 T cells subsets, less CD4 T cell activation, and proliferating PBs and memory B cells—was better captured by UMAP component 2. Immunotype 3, in which minimal lymphocyte activation response was observed, may represent ~20% of COVID-19 patients and is a potentially important scenario to consider for patients who may have failed to mount a robust antiviral T and B cell response. This UMAP integrated modeling approach could be improved in the future with additional data on other immune cell types and/or comprehensive data for circulating inflammatory mediators for all patients. Nevertheless, these findings provoke the idea of tailoring clinical treatments or future immune-based clinical trials to patients whose immunotype suggests greater potential benefit.

Respiratory viral infections can cause pathology as a result of an immune response that is too weak, resulting in virus-induced pathology, or too strong, leading to immunopathology (*58*). Our data suggest that the immune response of hospitalized COVID-19 patients may fall across this spectrum of immune response patterns, presenting as distinct immunotypes linked to clinical features, disease severity, and temporal changes in response and pathogenesis. This study provides a compendium of immune response data and an integrated framework to connect immune features to disease. By localizing patients on an immune topology map built on this dataset, we can begin to infer which types of therapeutic interventions may be most useful in specific patients.

## Materials and methods

### Patients, participants, and clinical data collection

Patients admitted to the Hospital of the University of Pennsylvania with a positive SARS-CoV-2 PCR test were screened and approached for informed consent within 3 days of hospitalization. Healthy donors (HDs) were adults with no prior diagnosis of or recent symptoms consistent with COVID-19. Normal reference ranges for HDs were the University of Pennsylvania clinical laboratory values shaded in green in Fig. 1B. Recovered donors (RDs) were adults with a prior positive COVID-19 PCR test by self-report who met the definition of recovery by the Centers for Disease Control and Prevention. HDs and RDs were recruited initially by word of mouth and subsequently through a centralized University of Pennsylvania resource website for COVID-19–related studies. Peripheral blood was collected from all participants. For inpatients, clinical data were abstracted from the electronic medical record into standardized case report forms. ARDS was categorized in accordance with the Berlin Definition, reflecting each individual’s worst oxygenation level and with physician adjudication of chest radiographs. APACHE III scoring was based on data collected in the first 24 hours of ICU admission or the first 24 hours of hospital admission for participants admitted to general inpatient units. Clinical laboratory data were abstracted from the date closest to that of research blood collection. HDs and RDs completed a survey about symptoms. After enrollment, the clinical team determined three patients to be COVID-negative and/or PCR false-positive. Two of these patients were classified as immunotype 3. In keeping with inclusion criteria, these individuals were maintained in the analysis. The statistical significance reported in Fig. 6K did not change when analysis was repeated without these three patients. All participants or their surrogates provided informed consent in accordance with protocols approved by the regional ethical research boards and the Declaration of Helsinki.

### Sample processing

Peripheral blood was collected into sodium heparin tubes (BD, catalog no. 367874). Tubes were spun [15 min, 3000 rpm, room temperature (RT)], and plasma was removed and banked. Remaining whole blood was diluted 1:1 with 1% RPMI (table S7) and layered into a SEPMATE tube (STEMCELL Technologies, catalog no. 85450) preloaded with lymphoprep (STEMCELL Technologies, catalog no. 1114547). SEPMATE tubes were spun (10 min, 1200×*g*, RT), and the PBMC layer was collected, washed with 1% RPMI (10 min, 1600 rpm, RT), and treated with ACK lysis buffer (5 min, ThermoFisher, catalog no. A1049201). Samples were filtered with a 70-μm filter, counted, and aliquoted for staining.

### Antibody panels and staining

Approximately 1 × 10^6^ to 5 × 10^6^ freshly isolated PBMCs were used per patient per stain. See table S7 for buffer information and table S8 for antibody panel information. PBMCs were stained with live/dead mix (100 μl, 10 min, RT), washed with fluorescence-activated cell sorting (FACS) buffer, and spun down (1500 rpm, 5 min, RT). PBMCs were incubated with 100 μl of Fc block (RT, 10 min) before a second wash (FACS buffer, 1500 rpm, 5 min, RT). Pellet was resuspended in 25 μl of chemokine receptor staining mix and incubated at 37°C for 20 min. After incubation, 25 μl of surface receptor staining mix was directly added, and the PBMCs were incubated at RT for a further 45 min. PBMCs were washed (FACS buffer, 1500 rpm, 5 min, RT) and stained with 50 μl of secondary antibody mix for 20 min at RT and then washed again (FACS buffer, 1500 rpm, 5 min, RT). Samples were fixed and permeabilized by incubating in 100 μl of Fix/Perm buffer (RT, 30 min) and washing in Perm Buffer (1800 rpm, 5 min, RT). PBMCs were stained with 50 μl of intracellular mix overnight at 4°C. The following morning, samples were washed (Perm Buffer, 1800 rpm, 5 min, RT) and further fixed in 50 μl of 4% paraformaldehyde (PFA). Before acquisition, samples were diluted to 1% PFA, and 10,000 counting beads were added per sample (BD, catalog no. 335925). Live/dead mix was prepared in phosphate-buffered saline (PBS). For the surface receptor and chemokine staining mix, antibodies were diluted in FACS buffer with 50% BD Brilliant Buffer (BD, catalog no. 566349). Intracellular mix was diluted in Perm Buffer.

### Flow cytometry

Samples were acquired on a five-laser BD FACS Symphony A5. Standardized SPHERO rainbow beads (Spherotech, catalog no. RFP-30-5A) were used to track and adjust photomultiplier tubes over time. UltraComp eBeads (ThermoFisher, catalog no. 01-2222-42) were used for compensation. Up to 2 × 10^6^ live PBMCs were acquired per sample.

### Luminex

PBMCs from patients were thawed and rested overnight at 37°C in complete RPMI (table S7). Flat-bottom plates with 96 wells were coated with 1 μg/ml of anti-CD3 (UCHT1, no. BE0231, BioXell) in PBS at 4°C overnight. The next day, cells were collected and plated at 1 × 10^5^ per well in 100 μl in duplicate. Anti-human CD28/CD49d (2 μg/ml) was added to the wells containing plate-bound anti-CD3 (Clone L293, 347690, BD). PBMCs were stimulated or left unstimulated for 16 hours and spun down (1200 rpm, 10 min), and supernatant (85 μl per well) was collected. Plasma from matched individuals was thawed on ice and spun (3000 rpm, 1 min) to remove debris, and 85 μl were collected in duplicate. Luminex assay was run according to manufacturer’s instructions, using a custom human cytokine 31-plex panel (EMD Millipore Corporation, SPRCUS707). The panel included EGF, FGF-2, eotaxin, sIL-2Ra, G-CSF, GM-CSF, IFN-α2, IFN-γ, IL-10, IL-12P40, IL-12P70, IL-13, IL-15, IL-17A, IL-1Ra, HGF, IL-1β, CXCL9/MIG, IL-2, IL-4, IL-5, IL-6, IL-7, CXCL8/IL-8, CXCL10/IP-10, CCL2/MCP-1, CCL3/MIP-1α, CCL4/MIP-1β, RANTES, TNF-α, and VEGF. Assay plates were measured using a Luminex FlexMAP 3D instrument (ThermoFisher, catalog no. APX1342).

Data acquisition and analysis were performed using xPONENT software (www.luminexcorp.com/xponent/). Data quality was examined on the basis of the following criteria: The standard curve for each analyte has a five-parameter *R*^2^ value > 0.95 with or without minor fitting using xPONENT software. To pass assay technical quality control, the results for two controls in the kit needed to be within the 95% confidence interval provided by the vendor for >25 of the tested analytes. No further tests were done on samples with results categorized as out-of-range low (<OOR). Samples with results that were out-of-range high (>OOR) or greater than the standard curve maximum value (SC max) were not tested at higher dilutions without further request.

### Intracellular stain after CD3/CD28 stimulation

Flat-bottom plates (96 wells) were coated with 1 μg/ml of anti-CD3 (UCHT1, no. BE0231, BioXell) in PBS at 4°C overnight. The next day, cells were collected and plated at 1 × 10^5^ per well in 100 μl with 1/1000 of GolgiPlug (BD, no. 555029). Anti-human CD28/CD49d (2 μg/ml) was added to the wells containing plate-bound anti-CD3 (Clone L293, 347690, BD). GolgiPlug-treated PBMCs were stimulated or left unstimulated for 16 hours, spun down (1200 rpm, 10 min), and stained for intracellular IFNγ.

### Longitudinal analysis D0 to D7 and patient grouping

To identify participants in which the frequency of specific immune cell populations increased, decreased, or stayed stable over time (D0 to D7), we used a previously published dataset (where data were available) to establish a standard range of fold change over time in a healthy cohort (*44*). A fold change greater than the mean fold change ± 2 standard deviations was considered an increase, less than this range was considered a decrease, and within this range was considered stable. Where these data were not available, a fold change from D0 to D7 of between 0.5 and 1.5 was considered stable. A fold change <0.5 was considered a decrease, and >1.5 was considered an increase. To eliminate redundant tests and maximize statistical power, the pairwise statistical tests shown in Fig. 5G were performed using fold change as a continuous metric, irrespective of the discrete up, stable, or down classification described above. Similarly, as shown in fig. S9G, pairwise association tests between changes in UMAP component coordinates and clinical data were performed using each difference value as a continuous metric, irrespective of the up, stable, or down classification.

### Correlation plots and heatmap visualization

Pairwise correlations between variables were calculated and visualized as a correlogram using R function *corrplot*. Spearman’s rank correlation coefficient (ρ) was indicated by square size and heat scale; significance was indicated by *P < 0.05, **P < 0.01, and ***P < 0.001; and a black box indicates a false-discovery rate (FDR) < 0.05. Heatmaps were created to visualize variable values using R function *pheatmap* or *complexheatmap*.

### Statistics

Owing to the heterogeneity of clinical and flow cytometric data, nonparametric tests of association were preferentially used throughout this study unless otherwise specified. Correlation coefficients between ordered features (including discrete ordinal, continuous scale, or a mixture of the two) were quantified by the Spearman rank correlation coefficient, and significance was assessed by the corresponding nonparametric methods (null hypothesis: ρ = 0). Tests of association between mixed continuous versus nonordered categorical variables were performed by unpaired Wilcoxon test (for *n* = 2 categories) or Kruskal-Wallis test (for *n* > 2 categories). Association between categorical variables was assessed by Fisher’s exact test. For association testing illustrated in heatmaps, categorical variables with more than two categories (e.g., ABO blood type) were transformed into binary “dummy” variables for each category versus the rest. All tests were performed in a two-sided manner, using a nominal significance threshold of *P* < 0.05 unless otherwise specified. When appropriate to adjust for multiple hypothesis testing, FDR correction was performed using the Benjamini-Hochberg procedure at the FDR < 0.05 significance threshold. Joint statistical modeling to adjust for confounding of demographic factors (age, sex, and race) when testing for association of UMAP components 1 and 2 with the NIH Ordinal Severity Scale was performed using ordinal logistic regression provided by the *polr* function of the R package *MASS*. Statistical analysis of flow cytometry data was performed using the R package *rstatix*. Other details, if any, for each experiment are provided within the relevant figure legends.

### High-dimensional data analysis of flow cytometry data

viSNE and FlowSOM analyses were performed on Cytobank (https://cytobank.org). B cells, non-naïve CD4 T cells, and non-naïve CD8 T cells were analyzed separately. viSNE analysis was performed using equal sampling of 1000 cells from each FCS file, with 5000 iterations, a perplexity of 30, and a theta of 0.5. For B cells, the following markers were used to generate the viSNE maps: CD45RA, IgD, CXCR5, CD138, Eomes, TCF-1, CD38, CD95, CCR7, CD21, KI67, CD27, CX3CR1, CD39, T-bet, HLA-DR, CD16, CD19 and CD20. For non-naïve CD4 and CD8 T cells, the following markers were used: CD45RA, PD-1, CXCR5, TCF-1, CD38, CD95, Eomes, CCR7, KI67, CD16, CD27, CX3CR1, CD39, CD20, T-bet, and HLA-DR. Resulting viSNE maps were fed into the FlowSOM clustering algorithm (*59*). For each cell subset, a new self-organizing map (SOM) was generated using hierarchical consensus clustering on the tSNE axes. For each SOM, 225 clusters and 10 or 15 metaclusters were identified for B cells and T cells, respectively.

To group individuals on the basis of B cell landscape, pairwise EMD values were calculated on the B cell tSNE axes for all COVID-19 D0 patients, HDs, and RDs using the *emdist* package in R, as previously described (*60*). Resulting scores were hierarchically clustered using the *hclust* package in R.

### Batch correction

During the sample-acquisition period, the flow panel was changed to remove one antibody. Batch correction was performed for samples acquired before and after this change to remove potential bias from downstream analysis. Because the primary flow features were expressed as a fraction of the parent population (falling in the 0-to-1 interval), a variance stabilizing transform (logit) was first applied to each data value prior to recentering the second panel to have the same mean as the first. After mean-centering, data were transformed back to the original fraction of parent scale by inverse transform. This procedure was applied separately to all 553 flow features annotated in the main text and supplemental data. Notably, this procedure avoids any batch-corrected feature values artificially falling outside of the original 0-to-1 range. After batch correction, neither UMAP component 1 nor component 2 had a statistically significant difference between panels by unpaired Wilcoxon test.

### Visualizing variation of flow cytometric features across the UMAP embedding space

A feature-weighted kernel density was computed across all COVID-19 patients and was displayed as a contour plot (Fig. 6G and fig. S8, A to D). Whereas traditional kernel density methods apply the same base kernel function to every point to visualize point density, in this case the base kernel function centered at each individual COVID-19 patient sample was instead weighted (multiplied) by the Z-transform (mean-centered and standard deviation–scaled) of the log-transformed input feature prior to computing the overall kernel density. This weighting procedure facilitated visualization of the overall feature gradients (from relatively low to high expression) across UMAP coordinates. independent of the different range of each input feature. A radially symmetric two-dimensional Gaussian was used as the base kernel function with a variance parameter of one-half, which was tuned to be sufficiently broad in order to smooth out local discontinuities and best visualize feature gradients.

### Definition of immunotype 3

To define COVID-19 patients with low or absent immune responses, classified as immunotype 3, the intersection of the bottom 50% of five different flow parameters was used: PB as percentage of B cells, KI67^+^ as percentage of non-naïve CD4 T cells, KI67^+^ as percentage of non-naïve CD8 T cells, HLA-DR^+^CD38^+^ as percentage of non-naïve CD4 T cells, and HLA-DR^+^CD38^+^ as percentage of non-naïve CD8 T cells. See fig. S10.

## The UPenn COVID Processing Unit

Zahidul Alam, Mary M. Addison, Katelyn T. Byrne, Aditi Chandra, Hélène C. Descamps, Yaroslav Kaminskiy, Jacob T. Hamilton, Julia Han Noll, Dalia K. Omran, Eric Perkey, Elizabeth M. Prager, Dana Pueschl, Jennifer B. Shah, Jake S. Shilan, Ashley N. Vanderbeck

University of Pennsylvania Perelman School of Medicine, Philadelphia, PA, USA.

## Supplementary Materials

science.sciencemag.org/content/369/6508/eabc8511/suppl/DC1 Figs. S1 to S11 Table S1 to S8

## Appendix

View/request a protocol for this paper from *Bio-protocol*.
